# Supplementary material for: 110 μm thin endo-microscope for deep-brain in vivo observations of neuronal connectivity, activity and blood flow dynamics
Source: Nat Commun. 2023 Apr 5;14:1897. doi: 10.1038/s41467-023-36889-z (PMC10076269; doi:10.1038/s41467-023-36889-z)
Supplement: Supplementary file 1 — Supplementary Information [file 41467_2023_36889_MOESM1_ESM.pdf]

Supplementary Information

Supplementary Figures

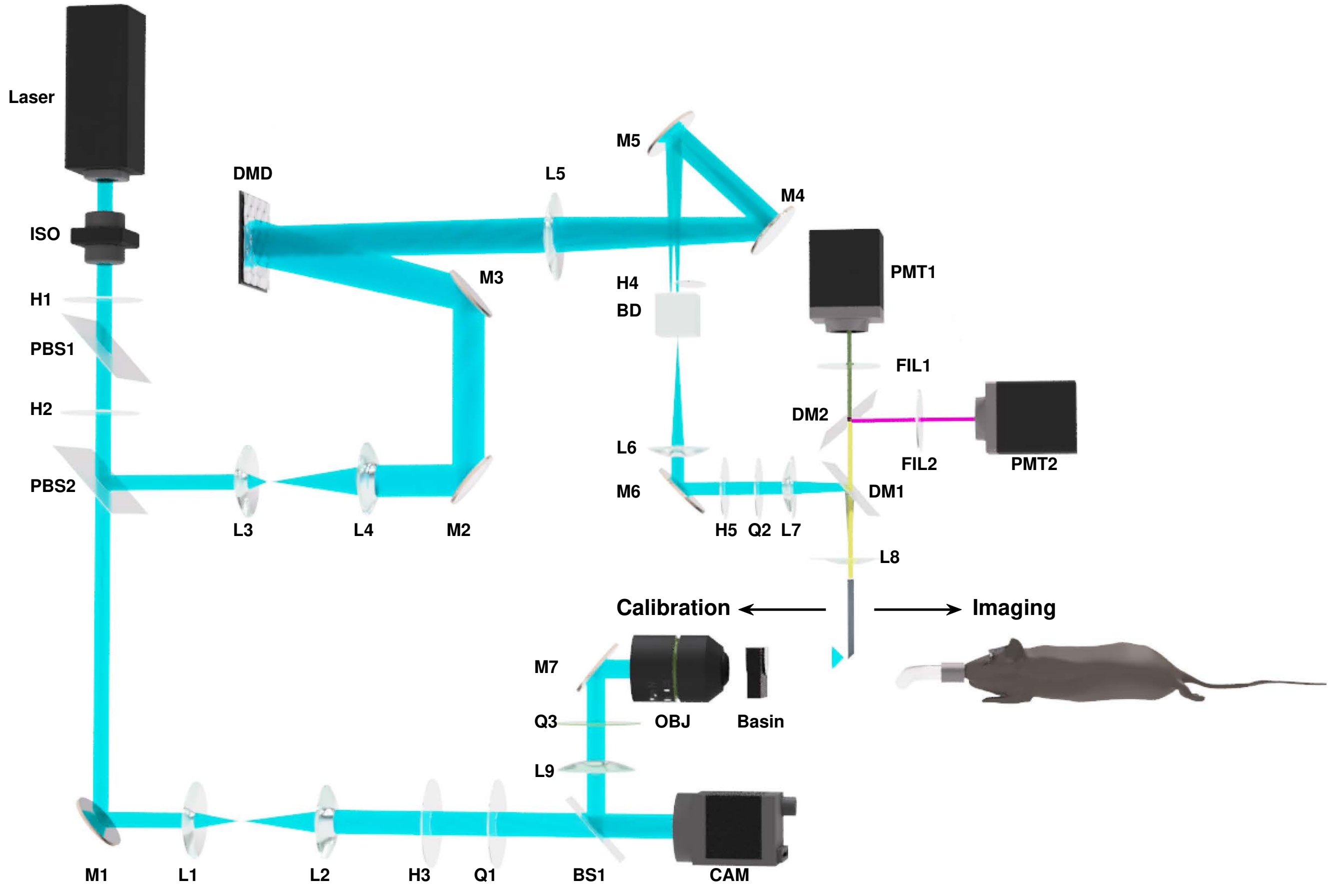

Fig. S1. The experimental geometry. BD: Calcite beam displacer, Thorlabs BD40. **BS1**: Beamsplitter plate, Thorlabs BSW10. **CAM**: Camera, Basler ace acA640-750um. **DM1**: Dichroic mirror, Chroma T495lpxru. **DM2**: Dichroic mirror, Semrock FF556-SDi01. **DMD**: Digital micromirror device, ViALUX V-7001. **FIL1**: Bandpass filter, Chroma ET510/20m. **FIL2**: Longpass filter, Thorlabs FEL0550. **H1–5**: Half-wave plate, Edmund Optics #46-551. **ISO**: Free-space optical isolator, Thorlabs IO-3-488-HP. **L1**: Lens, Thorlabs C240TMD-A ($f = 8$ mm). **L2**: Lens, Thorlabs AC254-300-A-ML ($f = 300$ mm). **L3**: Lens, Thorlabs C240TMD-A ($f = 8$ mm). **L4**: Lens, Thorlabs AC254-125-A-ML ($f = 125$ mm). **L5**: Lens, Thorlabs AC254-300-A-ML ($f = 300$ mm). **L6**: Lens, Thorlabs AC254-75-A-ML ($f = 75$ mm). **L7**: Lens, Thorlabs AC254-150-A-ML ($f = 150$ mm). **L8**: Lens, Thorlabs C240TMD-A ($f = 8$ mm). **L9**: Lens, Thorlabs AC254-150-A-ML ($f = 150$ mm). **Laser**: Laser, Coherent Sapphire 488 SF NX. **M1–7**: Dielectric mirror, Thorlabs BB1-E02. **OBJ**: Objective, Olympus 20X X-Apo 0.80NA/0.6WD. **PBS1,2**: Polarising beamsplitter, Thorlabs CCM1-PBS251/M. **PMT1,2**: GaAsP amplified photomultiplier tube, Thorlabs PMT2101/M. **Q1–3**: Quarter-wave plate, Thorlabs WPMQ10M-488.

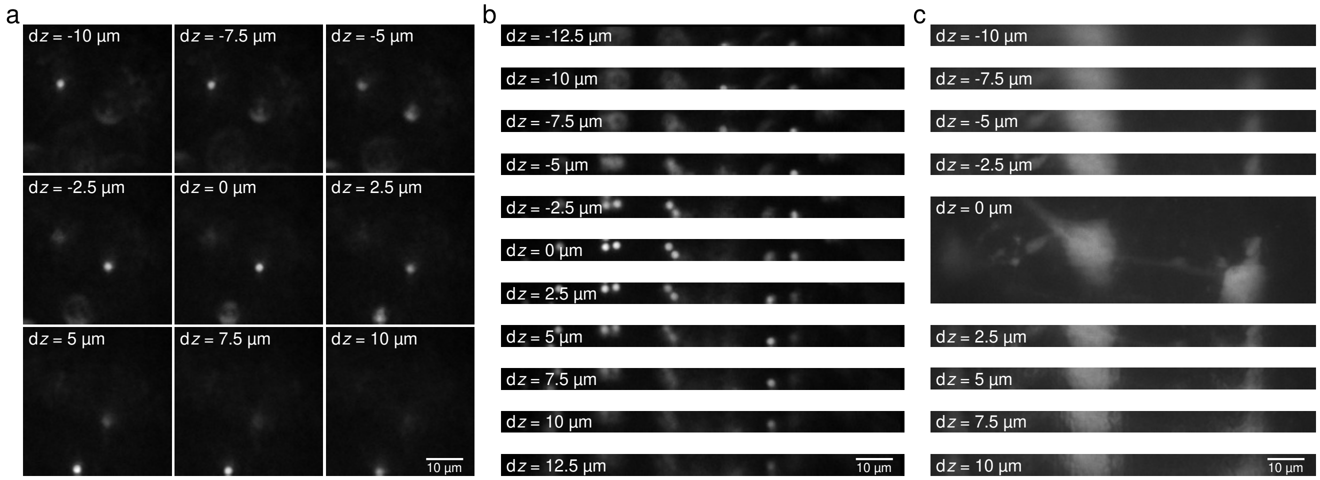

Fig. S2. Arrangements for volumetric scanning used in the presented experiments. a, Scanning arrangement used for volumetric time-lapse observations with stationary fibre. **b** and **c**, Scanning arrangement used while inserting the fibre. Extended central zone in **c** was included to improve the estimation of the probe insertion velocity.

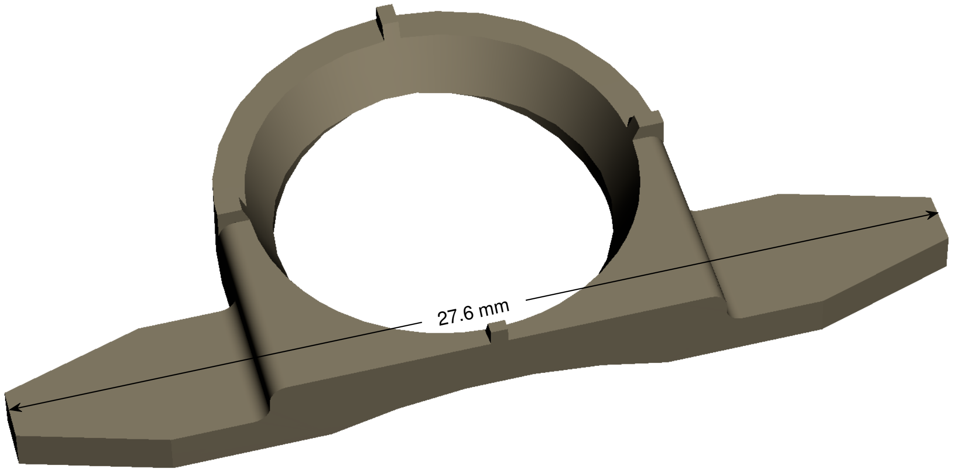

Fig. S3. Head plate used in *in-vivo* experiments.

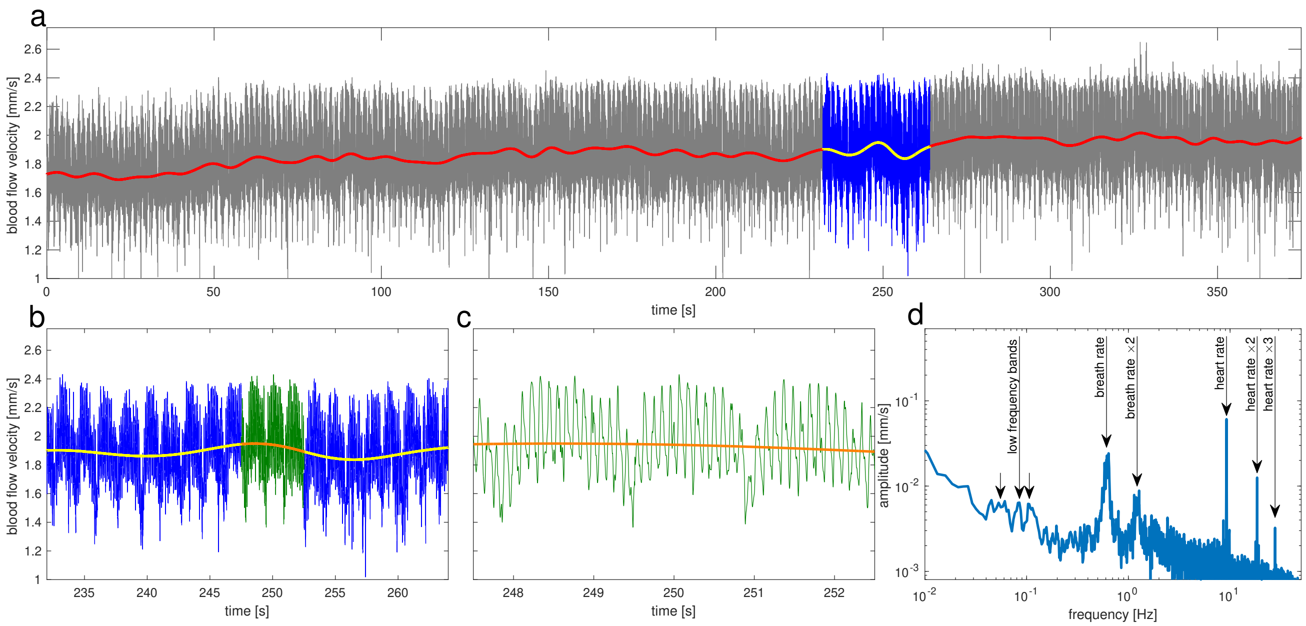

Fig. S4. Low-frequency oscillations of blood flow velocity. **a**, Record of blood flow velocity with and without low-pass filtering using a gaussian kernel with the width of 0.1 Hz. **b** and **c**, Zoomed-in regions of **a**. **d**, Frequency domain with the explanation of its features.

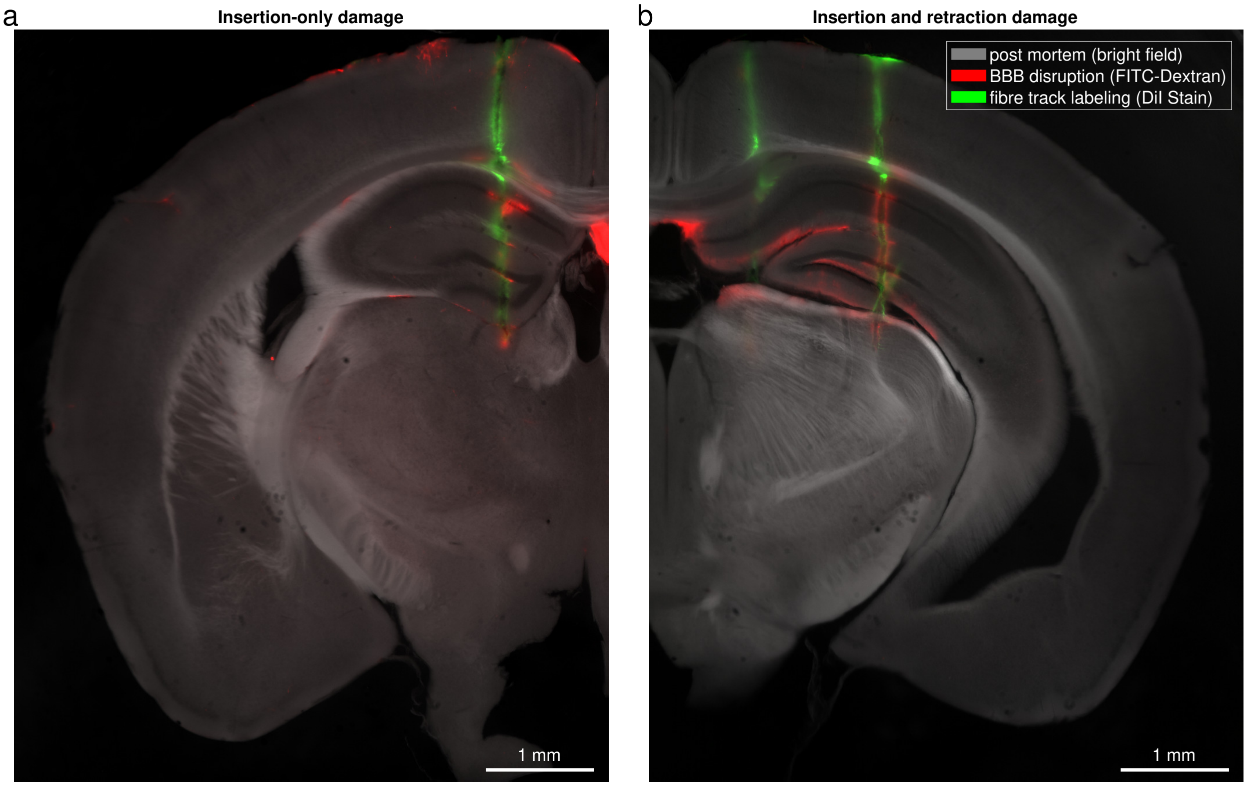

Fig. S5. Tissue damage assessment from post-mortem brain sections. As explained in Methods, FITC- Dextran was applied to the tested animals to visualise disruptions to blood-brain barrier. Prior to the animal was sacrificed, fibre probes were inserted in the same manner as in the imaging experiments. The probes were coated by DiI stain to visualise the trajectories in the tissue. **a**, The brain was harvested from the sacrificed animal and fixed prior to the probe retraction. **b**, The brain was harvested from the sacrificed animal and fixed after the probe was retracted. In this case, we see much more pronounced and spread bleeding than in the case of **a**.
