# Supplementary material for: 110 μm thin endo-microscope for deep-brain in vivo observations of neuronal connectivity, activity and blood flow dynamics
Source: Nat Commun. 2023 Apr 5;14:1897. doi: 10.1038/s41467-023-36889-z (PMC10076269; doi:10.1038/s41467-023-36889-z)
Supplement: Supplementary file 3 — Description of Additional Supplementary Files [file 41467_2023_36889_MOESM3_ESM.pdf]

Description of Additional Supplementary Files

File Name: Supplementary Movie 1
Description: Visualisation of volumetric record throughout the whole depth of the Thy1-GFP mouse brain. The scale bar indicates the distance of 50 mm.

File Name: Supplementary Movie 2
Description: Visualization of sub-cellular structures' dynamics within a single neurone. The scale bar indicates the distance of 10 mm.

File Name: Supplementary Movie 3
Description: Estimation of blood flow velocity. **a**, Original record of red blood cell traces. **b**, Enhanced record demanding the same mean value and standard deviation for each row. **c**, Morphed record windows (corresponding to that highlighted in **b**) counteracting the movement of the cells for the assumed velocity. **d**, Optimisation metric corresponding to morphed windows in c, polynomial fit across the minimum and its neighbours (dashed red curve) and the estimated value of velocity signified by red arrow. **e**, Reconstructed blood flow velocity.
