# Supplementary material for: 110 μm thin endo-microscope for deep-brain in vivo observations of neuronal connectivity, activity and blood flow dynamics
Source: Nat Commun. 2023 Apr 5;14:1897. doi: 10.1038/s41467-023-36889-z (PMC10076269; doi:10.1038/s41467-023-36889-z)
Supplement: Supplementary file 7 — Reporting Summary [file 41467_2023_36889_MOESM7_ESM.pdf]

nature portfolio

Corresponding author(s): Tomáš Čižmár

Last updated by author(s): Oct 20, 2022

Reporting Summary

Nature Portfolio wishes to improve the reproducibility of the work that we publish. This form provides structure for consistency and transparency in reporting. For further information on Nature Portfolio policies, see our Editorial Policies and the Editorial Policy Checklist.

Statistics

For all statistical analyses, confirm that the following items are present in the figure legend, table legend, main text, or Methods section.

n/a	Confirmed	
☐	☒	The exact sample size (*n*) for each experimental group/condition, given as a discrete number and unit of measurement
☒	☐	A statement on whether measurements were taken from distinct samples or whether the same sample was measured repeatedly
☒	☐	The statistical test(s) used AND whether they are one- or two-sided<br>*Only common tests should be described solely by name; describe more complex techniques in the Methods section.*
☒	☐	A description of all covariates tested
☒	☐	A description of any assumptions or corrections, such as tests of normality and adjustment for multiple comparisons
☐	☒	A full description of the statistical parameters including central tendency (e.g. means) or other basic estimates (e.g. regression coefficient) AND variation (e.g. standard deviation) or associated estimates of uncertainty (e.g. confidence intervals)
☒	☐	For null hypothesis testing, the test statistic (e.g. *F*, *t*, *r*) with confidence intervals, effect sizes, degrees of freedom and *P* value noted<br>*Give P values as exact values whenever suitable.*
☒	☐	For Bayesian analysis, information on the choice of priors and Markov chain Monte Carlo settings
☒	☐	For hierarchical and complex designs, identification of the appropriate level for tests and full reporting of outcomes
☒	☐	Estimates of effect sizes (e.g. Cohen's *d*, Pearson's *r*), indicating how they were calculated

Our web collection on statistics for biologists contains articles on many of the points above.

Software and code

Policy information about availability of computer code

Data collection	The data was collected in the form of voltage output from a photomultiplier tube (PMT) detector equiped with a build-in transimpedance amplifier controlled with LabVIEW drivers provided with the PMT.
Data analysis	Custom scripts written in MATLAB (version R2017b, tested also at R2021a) were used to process all data: The code for stitching of images acquired along the fibre track is available from https://doi.org/10.5281/zenodo.7524794. The code for processing of blood flow velocity is available from https://doi.org/10.5281/zenodo.7524782.

For manuscripts utilizing custom algorithms or software that are central to the research but not yet described in published literature, software must be made available to editors and reviewers. We strongly encourage code deposition in a community repository (e.g. GitHub). See the Nature Portfolio guidelines for submitting code & software for further information.

Data

Policy information about availability of data

All manuscripts must include a data availability statement. This statement should provide the following information, where applicable:

- Accession codes, unique identifiers, or web links for publicly available datasets
- A description of any restrictions on data availability
- For clinical datasets or third party data, please ensure that the statement adheres to our policy

The datasets generated and analysed during the presented studies are available in the Zenodo repository, https://doi.org/10.5281/zenodo.6598512.

Mouse Brain Atlas http://labs.gaidi.ca/mouse-brain-atlas has been used to navigate the imaging instrument into the desired location. Fig. 1b has been compiled using Allen Reference Atlas -- Adult Mouse -- Coronal Sections -- Average Template, available from atlas.brain-map.org.

Human research participants

Policy information about studies involving human research participants and Sex and Gender in Research.

Reporting on sex and gender	The study did not involve human research participants.
Population characteristics	N/A
Recruitment	N/A
Ethics oversight	N/A

Note that full information on the approval of the study protocol must also be provided in the manuscript.

Field-specific reporting

Please select the one below that is the best fit for your research. If you are not sure, read the appropriate sections before making your selection.

☒ Life sciences ☐ Behavioural & social sciences ☐ Ecological, evolutionary & environmental sciences

For a reference copy of the document with all sections, see nature.com/documents/nr-reporting-summary-flat.pdf

Life sciences study design

All studies must disclose on these points even when the disclosure is negative.

Sample size	No statistical method was used to define the sample size in the demonstrations of the imaging performance. Each demonstration was independently repeated for at least n=3. Even a single experimental session utilizing one animal yielded hundreds to thousands of high-quality images (technical replicates) showing clearly recognizable neuronal somata, processes, blood vessels and sub-cellular structures. Similarly in each experiment focusing on functional imaging, we have recorded abundant number of neurone spikes and red blood cell traces to demonstrate the system's capabilities.<br>This sample size was determined on the basis of previous studies with similar experiments, e.g., Biomedical Optics Express 9, 1492–1509 (2018) or Light-Science & Applications 7, 691 110 (2018). We consider this as trustworthy, beyond reasonable doubt and in line with the ethical (3R) principles.<br>The indicative demonstration of the impact of the 'straight-view' and 'side-view' probes on blood the flow velocity (Fig. 2l), was conducted with minimum sample size per single value of probe depth set to n=3. This obtained dataset is sufficient to verify that the insertion of the side-view probes affect the blood flow velocity measurements less than the straight-view probes when approaching the blood vessel (as the 99% confidence intervals do not overlap beyond the depth larger then d+15, the probability of null-hypothesis being correct is below 0.01%).
Data exclusions	For demonstration of different imaging modalities, high-quality images with cells or vessels in focus were selected based on visual inspection. Blood flow velocity data used for calculation of the velocity depression factor (Fig. 2l) were excluded only in the case when the imaged vessel became blurred (defocused) during imaging and during movement of the endoscopic probe in the tissue.
Replication	At the optimum performance, images acquired from different depth and locations of the brain were of comparable quality. All data for Fig. 2l, in which the blood flow during probe movements could be reconstructed in at least four positions (d, d+6, d+12 and d+18) were included. The unsuccessful attempts included mainly sessions in which the vessel got defocused and thus it was not possible to reconstruct the blood flow in all steps.
Randomization	The imaging sessions were sorted based on the imaging modality to be demonstrated (e.g. structural imaging, calcium imaging, blood flow imaging). No randomization was applied here. Measurements of the velocity depression factor (Fig. 2l) were sorted into groups based on the depth at which they were measured in respect to depth of the first measurement and based on the probe type. No randomization was applied here. No covariates were controlled for because the aim was to show the impact of the probe shape in general ('straight-view' vs. 'side-view') rather than studying the properties of the vessels themselves.
Blinding	This manuscript focuses on the presentation of a new technology. There is no information to be withheld from the participants of the experiments with which the presented data could have been affected. Example images for demonstration of the imaging performance of the endoscope were selected based on visual inspection to provide maximum utility in demonstrating the versatility of the new imaging technology. The velocity depression factors (Fig. 2l) were obtained by the same code regardless whether straight-view or side-view probe has been used (the code has been blind to the experimental conditions), all records in which the vessels remained in focus were processed and included in the statistics without any further discrimination.

Reporting for specific materials, systems and methods

We require information from authors about some types of materials, experimental systems and methods used in many studies. Here, indicate whether each material, system or method listed is relevant to your study. If you are not sure if a list item applies to your research, read the appropriate section before selecting a response.

Materials & experimental systems

n/a	Involved in the study
☐	☒ Antibodies
☒	☐ Eukaryotic cell lines
☒	☐ Palaeontology and archaeology
☐	☒ Animals and other organisms
☒	☐ Clinical data
☒	☐ Dual use research of concern

Methods

n/a	Involved in the study
☒	☐ ChIP-seq
☒	☐ Flow cytometry
☒	☐ MRI-based neuroimaging

Antibodies

Antibodies used	Anti-CD31, Thermofisher, eBioscience, Cat No 12-0311-81, Lot 2196690, Clone 390 at 1:5 dilution, total volume 50µl<br>Anti-CD34: Thermofisher, Invitrogen, Cat No MA5-17831, Lot WE3265594, Clone MEC14.7 at 1:5 dilution, total volume 50µl<br>Donkey anti-goat, Southern Biotech, Cat No 6420-0, Lot K5813-WI99X at 1:5 dilution, total volume 100µl<br>Goat anti-rat, Southern Biotech, Cat No 3050-09S, Lot C4208-X888P at 1:5 dilution, total volume 100µl
Validation	Our goal was to demonstrate the capabilty of the endoscope to image simultanously in two spectral channels. To this end we aimed at labeling of two distinct compartments with different fluorescent lables. While GFP was expressed in neurons under a Thy-1 promoter (mouse model), the vasculature was stained with phycoerythrin-labelled anti-mouse antibodies directed to endothelium molecules CD31 and CD34. The validation of vasculature labeling was performed in a 2-photon microscope, imaging dual color labeling with anti-CD31 and anti-CD34 in the red channel and intravenously injected fluorescein-dextran in the green channel.

Animals and other research organisms

Policy information about studies involving animals; ARRIVE guidelines recommended for reporting animal research, and Sex and Gender in Research

Laboratory animals	Mouse, Thy1-GFP line M (Stock No: 007788), floxed Ai6 (Stock No: 007906), B6J.ChAT-IRES-Cre (Dneo, Stock No: 031661), floxed Ai162D (Stock No: 031562), CaMK2a-CreERT2 (Stock No: 012362), wild-type C57BL/6J (Envigo), 8–24 weeks
Wild animals	The study did not involve wild animals.
Reporting on sex	Both male and female mice were used in this study. Due to the nature of the study, sex is not expected to affect the results (performance of the instrument) and was not considered in data analysis.
Field-collected samples	The study did not involve samples collected from the field.
Ethics oversight	Branch Commission for Animal Welfare of the Ministry of Agriculture of the Czech Republic (permissions no. 47/2020 and 49/2020)

Note that full information on the approval of the study protocol must also be provided in the manuscript.
